# Commentary: Beyond “TRIM” Benefits of β-Glucan by Blood Glucose and Lipid Balancing Potentials in Its Defense Against COVID-19

**DOI:** 10.3389/fimmu.2021.620658

**Published:** 2021-03-29

**Authors:** Nobunao Ikewaki, Vidyasagar Devaprasad Dedeepiya, Masaru Iwasaki, Samuel J. K. Abraham

**Affiliations:** ^1^ Department of Medical Life Science, Kyushu University of Health and Welfare, Nobeoka, Japan; ^2^ Institute of Immunology, Junsei Educational Institute, Nobeoka, Japan; ^3^ The Mary-Yoshio Translational Hexagon (MYTH), Nichi-In Centre for Regenerative Medicine (NCRM), Chennai, India; ^4^ School of Medicine, University of Yamanashi, Chuo, Japan; ^5^ Edogawa Evolutionary Laboratory of Science (EELS), Edogawa Hospital, Tokyo, Japan; ^6^ Life-systems through Immunology, Phylogeny and Evolution (LIPE) Division, GN Corporation Co. Ltd., Kofu, Japan

**Keywords:** COVID-19, trained immunity, β-glucan, blood glucose, fasting plasma glucose, lipid, coagulopathy, TRIM

## Introduction

The ‘hypothesis and theory’ article by Geller and Yan (1) is an eye-opener to the innate memory based Trained Immunity (TRIM) response offered by β-glucans in a prophylactic setting for immune-modulation to abrogate the symptoms in COVID-19, preventing morbidity and death.

Their review (1) begins with the evolutionary background of TRIM and moves on to its first recognition by the scientific community based on the non-specific immune response induced by the Bacille Calmette-Guérin (BCG) vaccine administered for Mycobacterium tuberculosis (TB). The multi-faceted TRIM offered by β-Glucans by virtue of their antiviral properties through production of IL-1β, TNF-α, and IFN-γ, induction of “central” memory by enabling production of a repertoire of innate cells from hematopoietic stem cells (HSC) in the bone marrow which migrate to peripheral tissues to generate peripheral memory and the epigenetic regulation through pathways of Dectin-1 activation (1) are elaborately narrated.

## Significance of Metabolic Regulation of TRIM

Geller and Yan (1) primarily focus on the anti-viral response of the metabolic regulation wherein modulation of the transcription of immunogenic genes occur by metabolic switch from oxidative phosphorylation toward aerobic glycolysis mediated through the AKT/mTOR/HIF1α pathway. Besides, it inhibits the LPS mediated upregulation of immune-responsive gene-1 (IRG-1) needed for itaconate generation. As high levels of itaconate and its derivatives inhibit key Type-I interferon production during viral infection, itaconate generation inhibited by this metabolic upregulation of β-glucans leads to increased Type I IFN response mediating anti-viral response against SARS-CoV-2 (1).

## Beyond “TRIM” Benefits of β-Glucans

Apart from the metabolic based anti-viral response explained by Geller and Yan (1) or the immune-modulation described by Rao et al. (2) in the defense against COVID-19, we would like to emphasize that regulation of blood glucose and lipid levels by β-Glucans as an indispensable tool of defense (3).

Diabetes, dyslipidemia and the immune system are inseparable and inter-connected factors of worsening the crisis post-COVID-19 infection. While high blood glucose levels lead to constant glucose recognition by C type lectin receptors which in turn lead to increased rate of inflammatory processes worsening the disease severity in COVID-19 (4), persistent chronic inflammation, decreased function of vascular endothelial cells and decreased immune function occurring in COVID-19 may actually underlie the pathogenesis of diabetes (5). Further, blood sugar levels increase the levels of clotting factors, causing endothelial dysfunction, enhanced platelet aggregation and activation, favoring a hypercoagulable pro-thrombotic state increase the risk of coagulopathy due to COVID-19 (6). High levels of low-density lipoprotein (LDL) lead to an increase in inflammatory gene expression and inflammasome activation through interaction with macrophages in atherosclerotic plaques causing worsening of the cytokine storm in COVID-19 (6). Low levels of high-density lipoprotein (HDL) are involved in the regulation of innate immune response and negatively regulates T-cell activation and the expression of inflammatory mediators in macrophages and dendritic cells (7). Dyslipidemia also causes endothelial dysfunction. With endothelial dysfunction induced coagulopathy having been identified to be the major cause of organ damage and death in COVID-19 patients (7), the contribution by high blood glucose and lipid levels cannot be ignored while planning prophylactic and therapeutic strategies to combat COVID-19 (8).

## β-Glucans in Normalization of Glucose and Lipid Levels in Clinical Studies

β-glucans such as the AFO-202 black yeast derived β-glucan have been shown to maintain the fasting plasma glucose (FPG) levels and bring down the high levels in diabetic patients to normal values in human clinical studies (9, 10) which has been hypothesized to be beneficial in the defense against COVID-19 (11). It is noteworthy that high FPG levels at admission has been proven as a strong independent predictor of poor outcomes in COVID-19 in several studies (12, 13). Further, the AFO-202 β-glucan has been shown to decrease LDL levels, very low-density lipoprotein (VLDL) levels and triglycerides to the normal range and increase lower HDL levels to reach their normal levels in human clinical studies (14).

## Mechanisms of Additional Benefits Offered by β-Glucans in TRIM and Metabolism

Immuno-metabolic circuits are critical in TRIM (15). Induction of glycolysis leads to immunotolerant monocytes as seen in sepsis by their effects on posttranslational modification of effector molecules such as glyceraldehyde 3-phosphate dehydrogenase (GAPDH) (16). Insulin resistance that occurs in obesity and diabetes facilitate TRIM in macrophages to acquire a state that has reduced responses to pathogens through metabolic and epigenetic changes. Insulin sensitization to reverse this state has been hypothesized to be utilized for inducing therapeutic TRIM response in conditions like cancer (16).

Apart from the above described beneficial effects that can contribute to the TRIM against COVID-19 by normalization of blood glucose and lipid levels, since β-glucan can reduce the insulin resistance which has been proven in human studies (17), a therapeutic TRIM response proving beneficial in COVID-19 will occur when β-glucans are employed as a prophylactic agents and the state of immunological tolerance which exists in individuals with obesity, diabetes and dyslipidemia can perhaps be reversed by these β-glucans (18).

## Conclusion

Therefore, in view of the excellent suggestion of TRIM induced by β-glucans as an “an efficient, low-cost and safe way” in a long term prophylactic setting applicable to different age groups of people and those with vacuous co-existing disease conditions in the defense against COVID-19 by Geller and Yan (1), we wish to reiterate that the metabolic effects of the β-glucans in terms of normalization of glucose levels, lipid levels and increase of insulin sensitivity significantly contributes to augment the TRIM response. Amidst the circumstances of a lack of definitive therapy or vaccine for COVID-19, Geller and Yan’s timely suggestion of undertaking clinical trials to confirm the efficacy of TRIM response induced by β-glucans from various sources (1) needs immediate acknowledgement by the scientific community, while duly recognizing their contribution to COVID-19 defense through the above described potential metabolic pathways and their validation, for an efficient fight against this devastating pandemic.

## Author Contributions

NI and SA contributed to conception and design. SA drafted the manuscript. VD and MI performed critical revision of the manuscript. All authors contributed to the article and approved the submitted version.

## Conflict of Interest

SA is a shareholder in GN Corporation, Japan, which in turn is a shareholder in the manufacturing company of the AFO 202 Beta Glucan product and an applicant to several patents of relevance.

The remaining authors declare that the research was conducted in the absence of any commercial or financial relationships that could be construed as a potential conflict of interest.
